# 235. Investigation of the relationship between the number of positive blood cultures and patient prognosis

**DOI:** 10.1093/ofid/ofad500.308

**Published:** 2023-11-27

**Authors:** Satoru Sekiya, Ryo Hasegawa, Koko Shibutani, Mihye Lee, Nobuyoshi Mori

**Affiliations:** St Luke's international hospital, Chuo-ku, Tokyo, Japan; Akita University Hospital, akita, Akita, Japan; St Luke's international hospital, Chuo-ku, Tokyo, Japan; Graduate School of Public Health, St. Luke’s International University, Chuo-ku, Tokyo, Japan; St. Luke's International Hospital, Tokyo, Tokyo, Japan

## Abstract

**Background:**

The relationship between the number of positive blood cultures (NPBCs) and clinical outcomes has not been well-studied. The purpose of this study was to assess the impact of NPBCs (one set or two) on clinical outcomes.

**Methods:**

We conducted a retrospective study comparing the clinical features of two sets of positive blood cultures (NPBCs=2) and one set of positive blood culture (NPBCs=1) in adult patients with bacteremia and fungemia from 2004 to 2021 at a tertiary hospital in Tokyo, Japan. We focused on four pathogens commonly encountered in clinical practice, including *Staphylococcus aureus* (*S. aureus*), *Streptococci*, gram-negative rods (GNR) (*Enterobacterales* and *Pseudomonas aeruginosa*), and *Candida* species, as the true causative pathogens in bacteremia and fungemia. The primary outcome was in-hospital mortality, and secondary outcomes were ICU admission and use of vasopressor drugs.

**Figure:**
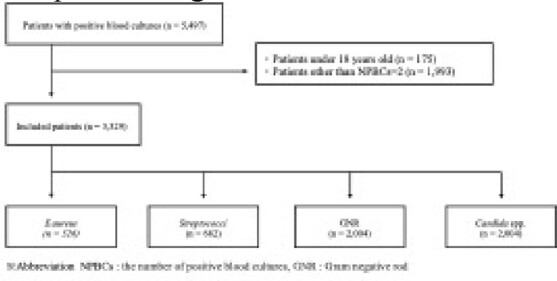

**Results:**

In univariate analysis, NPBCs=2 for *S. aureus* was associated with vasopressor use, and NPBCs=2 for *Streptococci* was linked to a higher rate of ICU admission. However, these differences were not significant in multivariate analysis. Interestingly, NPBCs=2 for *S. aureus* had a positive impact on in-hospital mortality compared with NPBCs=1 (OR 0.574, 95%CI 0.376–0.877, p < 0.05). NPBCs for *Candida* species did not affect clinical outcomes. Furthermore, NPBCs=2 for GNR was associated with vasopressor use (OR 1.62, 95%CI 1.27–2.07, p < 0.05) in both univariate and multivariate analysis. We performed logistic regression analysis using the five items with the highest clinical significance: NPBCs=2, serum Cr > 1.2 mg/dL, Hb < 12 g/dL, heart rate > 90 beats/min, and platelet count < 150,000/μL. Based on the β coefficient, we assigned scores to each item and created a STAT-2 (S: Serum Cr, T: Thrombocytopenia, A: Anemia, T: Tachycardia, 2: NPBCs=2) score for the use of hypertensive drugs for GNR. The receiver operating characteristic (ROC) curve for the STAT-2 score with a cutoff of 6 points or higher had an area under the curve (AUC) of 0.763. The specificity was 90.8%, with a positive likelihood ratio of 4.13 (95% CI 3.40-5.02).

**Conclusion:**

Our study suggests that NPBCs can predict clinical outcomes in patients with certain pathogenic infections.

**Disclosures:**

**All Authors**: No reported disclosures

